# Influence of prior myocardial infarction on outcome in patients with ischaemic HFrEF: insights from the EVIdence based TreAtment in Heart Failure (EVITA-HF) registry

**DOI:** 10.1007/s00392-024-02455-w

**Published:** 2024-05-08

**Authors:** Tobias Heer, Uwe Zeymer, Matthias Hochadel, Lutz Frankenstein, Matthias Pauschinger, Rainer Hambrecht, Oliver Bruder, Michael Böhm, Lars S. Maier, Ralf Zahn, Jochen Senges

**Affiliations:** 1 Department of Cardiology, München Klinik Neuperlach, Academic Teaching Hospital, LMU University of Munich, Oskar-Maria-Graf-Ring 51, 81737 Munich, Germany; 2 Department of Cardiology, Klinikum der Stadt Ludwigshafen am Rhein, Ludwigshafen, Germany; 3 Stiftung Institut für Herzinfarktforschung, Ludwigshafen, Germany; 4 Department of Internal Medicine III, Universitätsklinikum Heidelberg, Heidelberg, Germany; 5 Med. Klinik 8 - Kardiologie, Universitätsklinik der Paracelsus Medizinischen Privatuniversität, Klinikum Nürnberg, Nuremberg, Germany; 6 Department of Internal Medicine II, Krankenhaus Links der Weser, Bremen, Germany; 7 Department of Cardiology and Angiology, Elisabeth-Krankenhaus Essen, Essen, Germany; 8 Department of Cardiology, Universitätsklinikum des Saarlandes, University of Saarland, Homburg/Saar, Germany; 9 Department of Internal Medicine II, Universitätsklinikum Regensburg, Regensburg, Germany

**Keywords:** Prior myocardial infarction, Outcome, Health status, Ischaemic HFrEF

## Abstract

**Background:**

There is scarce information about the influence of prior myocardial infarction (pMI) on outcomes in patients (pts) with ischaemic HFrEF. We analysed data from the EVIdence based TreAtment in Heart Failure (EVITA-HF) registry.

**Methods:**

EVITA-HF comprises web-based case report data on demography, diagnostic measures, adverse events and 1-year follow-up of patients hospitalized for chronic heart failure ≥ 3 months (CHF) and an ejection fraction ≤ 40%. In the present study, we focused on the outcomes of pts with and without pMI in ischaemic HFrEF.

**Results:**

Between February 2009 and November 2015, a total of 2075 consecutive pts with ischaemic HFrEF were included from 16 centres in Germany. A total of 81.2% were male, and the mean age was 71 years. A total of 61.5% of the pts with ischaemic HFrEF had a history of pMI. These pts were treated less often with PCI (20.0 vs. 31.0%, *p* < 0.001) or CABG (3.8 vs. 7.7%, *p* < 0.001). They more often received an ICD (40.9 vs. 28.7%, *p* < 0.001), but less often a CRT-D (11.3 vs. 19.4%, *p* < 0.001). After multivariate adjustment, pts with pMI had a greater all-cause mortality after 1 year than those without pMI (hazard ratio 1.4; 95% CI, 1.10–1.79, *p* = 0.007). The combined endpoint of death, resuscitation or ICD shock after 1 year was greater in patients with pMI (20.8 vs. 16.4%, *p* = 0.03). Mobility was more often reduced in pts with pMI (46.8% vs. 40.1%, *p* = 0.03), and overall health status was more frequently worse in patients with pMI than in those 12 months ago (23.1 vs. 15.9%, *p* = 0.01). More than a quarter of the pts with ischaemic HFrEF were anxious or depressive.

**Conclusion:**

pMI in patients with CHF and ischaemic HFrEF was associated with increased mortality, increased event rates, and worsened health status. Hence, the subgroup of pts with ischaemic HFrEF and pMI is at higher risk and deserves special attention.

**Graphical abstract:**

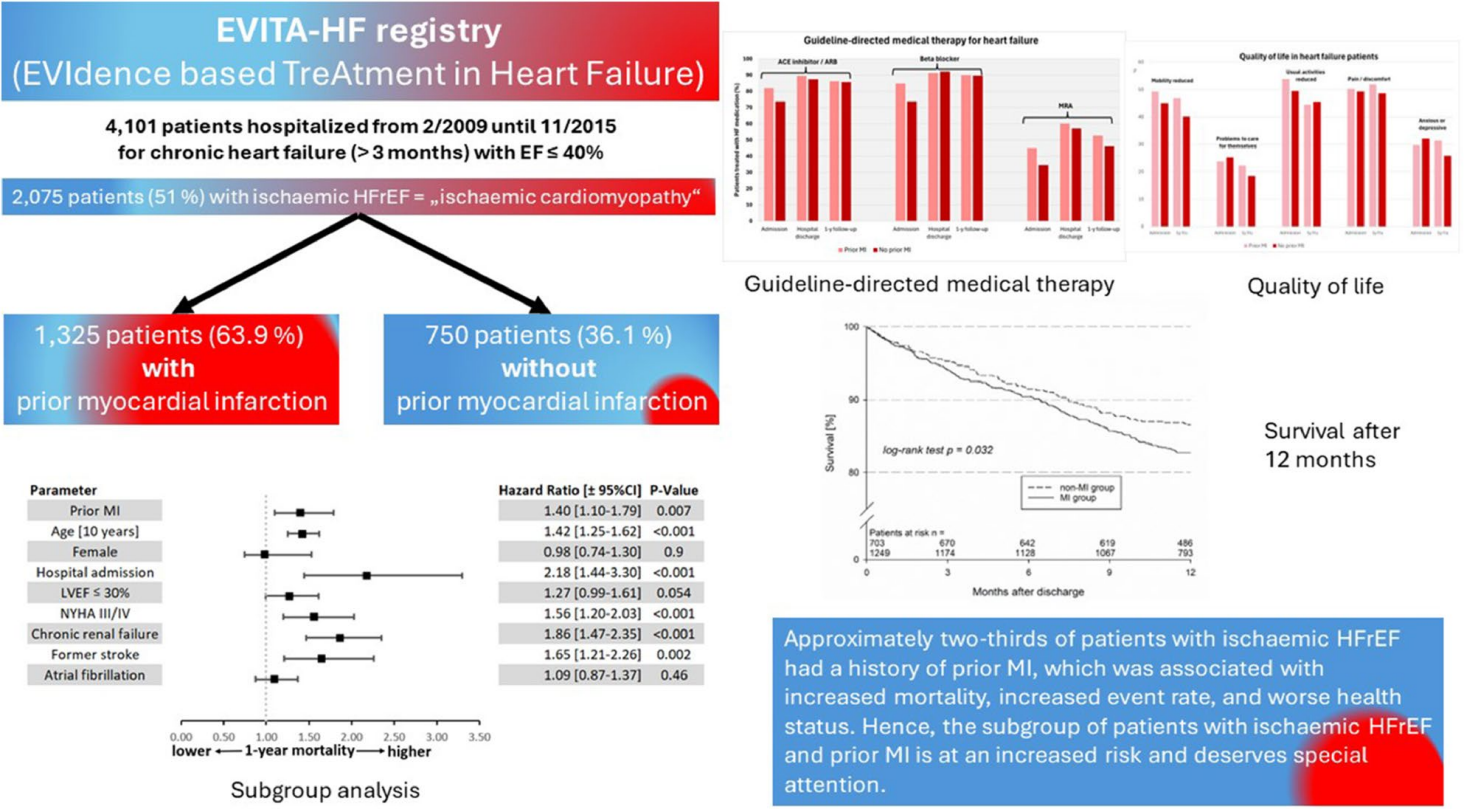

## Introduction

The incidence and prevalence of heart failure (HF) have been growing over the last several decades [1–6]. HF of origin has become increasingly common because of the improved survival of patients with acute myocardial infarction (AMI) [1, 7, 8]. However, knowledge about the clinical differences and their impact on the prognosis of patients with ischaemic HFrEF is limited [1, 9]. Treatment of AMI and HF has improved substantially. Thus, the population at risk for sudden cardiac death (SCD) may also have been altered. However, data from previous trials may no longer be applicable [1, 10]. The pathophysiological substrate of ischaemic HFrEF is heterogeneous, varying from predominantly hibernating myocardium to irreversible scarring [7]. Registries have been developed to improve the quality of care and outcomes for patients with HF [11]. The aim of the present subanalysis of the EVIdence based TreAtment in Heart Failure (EVITA-HF) registry [12] was to analyse the effect of prior MI on the prognosis of patients with ischaemic HFrEF.

## Methods

**Fig. 1:**
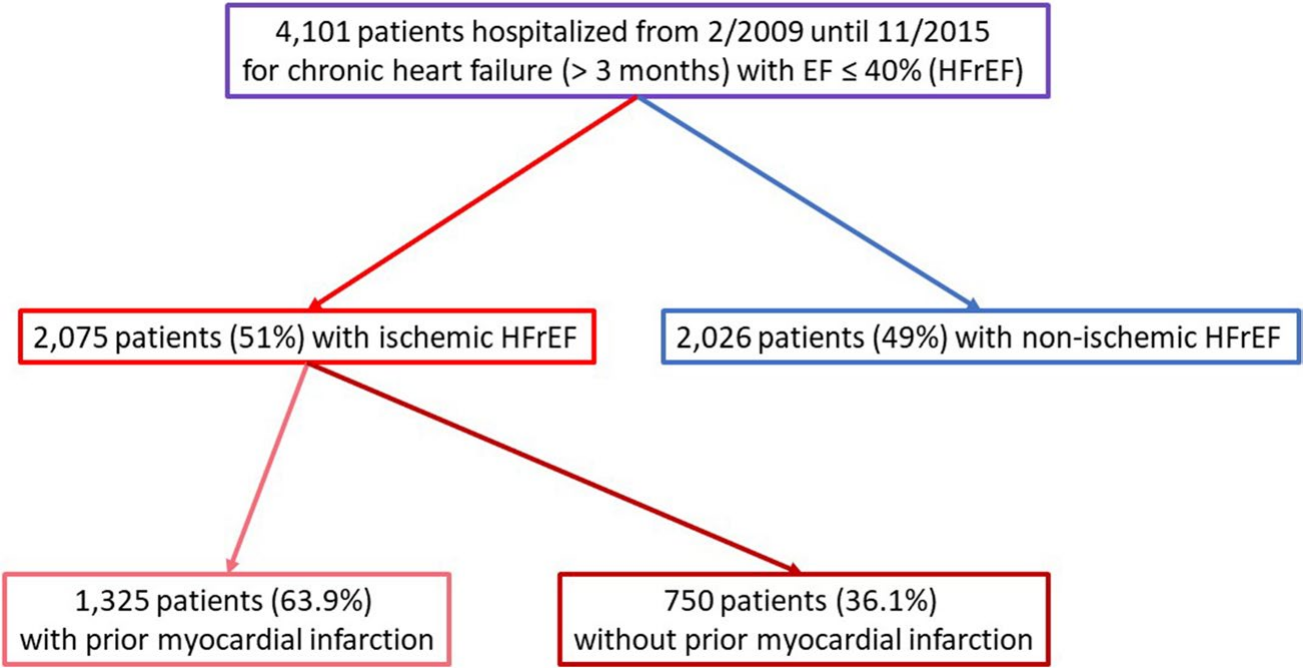
Composite of EVITA-HF patients and the selection criteria for the present analysis

EVITA-HF is a registry of HF patients from 16 German tertiary care centres which includes the whole spectrum of diagnostic and treatment modalities for HF (Fig. 1). Patients were hospitalized in one of the 16 participating hospitals and had to be included consecutively [12]. As described by von Scheidt et al. [13], the inclusion criteria were chronic HF ≥ 3 months and a documented ejection fraction ≤ 40%. The diagnosis of ischaemic HFrEF was made by experienced cardiologists considering a history of myocardial infarction, and/or the electrocardiogram (any pathologic Q waves in relevant anterior or inferior leads), and/or the echocardiogram (any relevant akinesia consistent with a former myocardial infarction with explains the reduced LV function), or the coronary angiography (occlusion of any large coronary artery without relevant collaterals; only if available, was not demanded), or the cardiac magnetic resonance tomography (any relevant ischaemic scar consistent with former myocardial infarction; only if available, was not demanded). The exclusion criterion consisted of patients aged younger than 18 years or who provided no consent. Patient data were collected using a web-based electronic case report form (eCRF). Data management was performed at the Institut für Herzinfarktforschung Ludwigshafen, Germany. The registry was approved by the ethics committees of the participating centres [12]. Details of the methods used in the EVITA-HF trial were published previously [12, 13]. The EVITA-HF trial started in January 2009 and included 4101 patients as of November 2015. Baseline information concerning demographics, medical history, clinical evaluation, and diagnostics as well as pharmacological and non-pharmacological treatment, quality of life and adverse events during index hospitalization were gathered by eCRF. One-year follow-up was performed by phone calls and/or contact by the centre or general practitioner. Follow-up data consisted of vital status, adverse events and interventions since index discharge and current health status, pharmacological treatment and quality of life. One-year follow-up was defined as status obtained between 300 and 450 days after index discharge.

Here we present the data of all patients with ischaemic HFrEF. The Minnesota Living With Heart Failure Questionnaire (MLWHFQ) was used to evaluate the quality of life of patients with HF. The questionnaire comprises 21 questions about several physical, emotional and socioeconomic aspects that can adversely affect a patient’s life.

### Statistical analysis

The patient population was described by absolute numbers and percentages with respect to categorical variables and by medians with quartiles for continuous variables. The distributions of dichotomous variables were compared between patient groups by the Pearson chi-square test, and odds ratios (ORs) with 95% confidence intervals (CIs) were calculated. The Mann–Whitney test was used for comparisons of categorical or ordinal variables. One-year survival and event-free survival after index discharge were analysed using the product-limit method and the log-rank test. The results are demonstrated in Kaplan–Meier curves for patients with vs. without prior MI in total. Corresponding hazard ratios were calculated via Cox regression models and were unadjusted or adjusted for the clinically relevant risk factors age, sex, LVEF ≤ 30%, NYHA III/IV on admission, chronic kidney disease and atrial fibrillation. The interaction between the two subgroups was assessed by the Wald test. All tests performed were two-sided and a *p* value ≤ 0.05 was used to indicate statistical significance. The computations were performed using the SAS system (release 9.4, SAS Institute, Inc., Cary, NC).

## Results

### Baseline data

The baseline demographic data and comorbidities of 2075 EVITA-HF patients with ischaemic HFrEF are given in Table 1. The mean age was 71 years for patients with a history of MI and 72 years for patients without prior MI. Patients with a history of MI more often underwent prior coronary revascularization procedures including PCI and CABG (78.6 vs. 56.0%, *p* < 0.001). More patients had hypertension (79.0 vs. 75.1%, *p* = 0.04), but had atrial fibrillation less often on admission (31.4 vs. 39.5%, *p* < 0.001). Patients with diabetes and prior MI were likely to be on insulin (44.2 vs. 36.2%, *p* = 0.02). Patients with prior MI were more often previously hospitalized for HF (66.7 vs. 58.2%, *p* = 0.003), more often had an ICD implanted (30.2 vs. 16.4%, *p* < 0.001), and had less often had a pacemaker (4.1 vs. 7.7%, *p* < 0.001). On hospital admission, patients with prior MI more often had an existing therapy with ACE inhibitors or angiotensin receptor blockers (82.0 vs. 73.6%, *p* < 0.001), beta-blockers (84.7 vs. 73.6%, *p* < 0.001), mineralocorticoid receptor blockers (44.9 vs. 34.6%, *p* < 0.001) or diuretics (75.9 vs. 65.8%, *p* < 0.001). Clinical and technical findings at index presentation are given in Table 2.

**Table 1:** Baseline demographic and comorbidity data of characteristics of 2075 EVITA-HF patients with ischaemic HFrEF

	History of myocardial infarction	No history of myocardial infarction	*p* value	Odds ratio (95% CI)
*n*, (%)	1325 (63.9)	750 (36.1)		
Age, years (median)	71 (61, 77)	72 (63, 78)		
Male gender, (%) *n*	81.2 (1076/1325)	82.1 (616/750)	0.60	0.94 (0.75–1.19)
LV EF (median)	30 (25, 35)	30 (25, 35)	0.95	
EF ≤ 35, % (*n*/total *n*)	82.4 (1079/1310)	82.0 (607/740)	0.85	1.02 (0.81–1.30)
Any prior revascularization (PCI and/or CABG), % (*n*/total *n*)	78.6 (992/1262)	56.0 (401/716)	< 0.001	2.89 (2.36–3.52)
Prior PCI, % (*n*/total *n*)	59.7 (754/1262)	38.4 (275/716)	< 0.001	2.38 (1.97–2.87)
Prior CABG, % (*n*/total *n*)	35.3 (445/1262)	29.9 (214/716)	0.02	1.28 (1.05–1.56)
Prior valve surgery/intervention, % (*n*/total *n*)	4.3 (54/1262)	7.7 (55/715)	0.001	0.54 (0.36–0.79)
Atrial fibrillation, % (*n*/total *n*)	31.4 (416/1325)	39.5 (296/750)	< 0.001	0.70 (0.58–0.85)
Hypertension, % (*n*/total *n*)	79.0 (1047/1325)	75.1 (563/750)	0.04	1.25 (1.01–1.55)
Diabetes mellitus	44.1 (584/1325)	43.2 (324/750)	0.70	1.04 (0.86–1.24)
-On insulin	44.2 (257/582)	36.2 (117/323)	0.02	1.39 (1.05–1.84)
Stroke, % (*n*/total *n*)	9.1 (121/1325)	9.2 (69/750)	0.96	0.99 (0.73–1.35)
Peripheral artery disease, % (*n*/total *n*)	13.4 (178/1324)	13.9 (104/750)	0.79	0.96 (0.74–1.25)
Chronic kidney disease, % (*n*/total *n*)	34.7 (460/1325)	33.2 (249/750)	0.48	1.07 (0.89–1.29)
Previously hospitalized for HF, % (*n*/total *n*)	66.7 (584/875)	58.2 (234/402)	0.003	1.44 (1.13–1.84)
Implanted device (ICD, CRT-D, CRT-P, PM), % (*n*/total *n*)	41.5 (550/1325)	31.4 (235/748)	< 0.001	1.55 (1.28–1.87)
Pacemaker, % (*n*/total *n*)	4.1 (54/1323)	7.7 (57/745)	< 0.001	0.51 (0.35–0.75)
ICD, % (*n*/total *n*)	30.2 (400/1323)	16.4 (122/745)	< 0.001	2.21 (1.76–2.78)
CRT-D, % (*n*/total *n*)	6.8 (90/1323)	6.7 (50/745)	0.937	1.01 (0.71–1.45)

*CMP* cardiomyopathy, *PCI* percutaneous coronary intervention, *CABG* coronary artery bypass graft, *PM* pacemaker, *ICD* implantable cardioverter-defibrillator, *CRT* cardiac resynchronization therapy

**Table 2:** Clinical findings at index presentation

	History of myocardial infarction *n* = 1161 (64.2%)	No history of myocardial infarction *n* = 647 (35.8%)	*p* value	Odds ratio (95% CI)
*n* (%)	1325 (63.9)	750 (36.1)		
Inpatient stay, % (*n*)	75.3 (998/1325)	83.5 (626/750)	< 0.001	0.60 (0.48–0.76)
Outpatient clinic, % (*n*)	24.7 (327/1325)	16.5 (124/750)	< 0.001	1.65 (1.31–2.08)
BMI (kg/m^2^)*	27.1 (24.4, 30.9)	27.2 (24.3, 30.4)	0.60	
Systolic blood pressure, mmHg	120 (110, 138)	130 (110, 140)	< 0.001	
Diastolic blood pressure, mmHg	70 (60, 80)	75 (67, 80)	< 0.001	
NYHA functional class II, % (*n*/total *n*)	34.3 (454/1325)	31.7 (238/750)		
NYHA functional class III, % (*n*/total *n*)	42.3 (560/1325)	44.4 (333/750)		
NYHA functional class IV, % (*n*/total *n*)	13.7 (181/1325)	15.6 (117/750)		
NYHA III +	55.9 (741/1325)	60.0 (450/750)	0.07	0.85 (0.71–1.01)
ACE inhibitor/ARB, % (*n*/total *n*)	82.0% (1087/1325)	73.6% (551/749)	< 0.001	1.64 (1.32–2.03)
Beta-blocker, % (*n*/total *n*)	84.7% (1121/1324)	73.6% (551/749)	< 0.001	1.98 (1.59–2.47)
MRA, % (*n*/total *n*)	44.9% (595/1324)	34.6% (259/749)	< 0.001	1.54 (1.28–1.86)
Diuretics, % (*n*/total *n*)	75.9% (1006/1325)	65.8% (493/749)	< 0.001	1.64 (1.35–1.99)
Quality of life, MLWHFQ score**	35 (20, 51)	37 (21, 51)		

^*^Median (quartiles), *BMI* body mass index, *NYHA* New York Heart Association, *LVEF* left ventricular ejection fraction, **documented in 36% of patients only due to later introduction in June 2011

### Hospital course and treatments

Table 3 shows the clinical course and interventions of the hospitalized patients. There was no difference in hospital mortality, nonfatal MI, nonfatal stroke or the combination of death, MI or stroke between patients with and without prior MI (Table 3). Patients with prior MI were less likely to be resuscitated in the hospital (6.5 vs. 2.0%, *p* = 0.02) and treated less often with PCI (20.0 vs. 31.0%, *p* < 0.001) or CABG (3.8 vs. 7.7%, *p* < 0.001). There was no difference in the rate of device implantations/revisions between the two groups and there was no difference in the rate of electrical cardioversion (Table 3).

**Table 3:** Hospital course—complications and interventions

	History of myocardial infarction	No history of myocardial infarction	*p* value	Odds ratio (95% CI)
*n*, (%)	998 (61.5)	626 (38.5)		
Complications				
Hospital mortality, % (*n*/total *n*)	2.5 (25/997)	1.4 (9/626)	0.14	1.76 (0.82–3.80)
Nonfatal myocardial infarction, % (*n*/total *n*)	2.0 (13/659)	4.0 (13/329)	0.07	0.49 (0.22–1.07)
Nonfatal stroke, % (*n*/total *n*)	0.8 (5/659)	0.0 (0/329)	0.11	
Death, MI, stroke (MACCE), % (*n*/total *n*)	5.0 (33/660)	5.1 (17/331)	0.93	0.97 (0.53–1.77)
Resuscitation, % (*n*/total *n*)	2.0 (6/293)	6.5 (10/155)	0.02	0.30 (0.11–0.85)
ICD shock, % (*n*/total *n*)	1.4 (4/293)	1.3 (2/155)	0.95	1.06 (0.19–5.85)
Interventions				
PCI, % (*n*/total *n*)	20.0 (199/994)	31.0 (193/622)	< 0.001	0.56 (0.44–0.70)
CABG, % (*n*/total *n*)	3.8 (38/994)	7.7 (48/622)	< 0.001	0.48 (0.31–0.74)
Device implantation or revision, % (*n*/total *n*)	20.5 (204/996)	23.5 (146/622)	0.16	0.84 (0.66–1.07)
Cardioversion, % (*n*/total *n*)	5.0% (50/994)	7.1% (44/622)	0.09	0.70 (0.46–1.06)

At discharge, there were only minor differences in medication use between the two groups with a high percentage of patients receiving guideline-directed heart failure therapy in both groups (Table 4). Statins were more often prescribed in patients with prior MI (86.5 vs. 77.4%, *p* < 0.001). Patients with prior MI more often had an implanted device (53.1 vs. 46.1%, *p* = 0.004).

**Table 4:** Medication and device status at discharge in patients discharged alive (*n* = 2040)

	History of myocardial infarction	No history of myocardial infarction	*p* value	Odds ratio (95% CI)
*n*, (%)	1299 (63.7)	741 (36.3)		
ACE inhibitor/ARB, % (*n*/total *n*)	89.4 (1157/1294)	87.3 (645/739)	0.15	1.23 (0.93–1.63)
Beta-blocker, % (*n*/total *n*)	91.3 (1179/1292)	92.2 (681/739)	0.48	0.89 (0.64–1.24)
MRA, % (*n*/total *n*)	60.1 (777/1293)	57.4 (424/739)	0.231	1.12 (0.93–1.34)
Diuretics, % (*n*/total *n*)	83.8 (1085/1295)	80.5 (595/739)	0.06	1.25 (0.99–1.58)
Statin, % (*n*/total *n*)	86.5 (1120/1295)	77.4 (572/739)	< 0.001	1.87 (1.48–2.36)
ASS, % (*n*/total *n*)	93.6 (817/873)	94.8 (459/484)	0.35	0.79 (0.49–1.29)
Clopidogrel, % (*n*/total *n*)	41.1 (359/873)	44.6 (216/484)	0.211	0.87 (0.69–1.08)
Prasugrel, % (*n*/total *n*)	5.4 (28/518)	5.2 (17/330)	0.87	1.05 (0.57–1.95)
Ticagrelor, % (*n*/total *n*)	6.3 (14/223)	8.7 (15/172)	0.36	0.70 (0.33–1.49)
Oral anticoagulants, % (*n*/total *n*)	37.7 (488/1295)	39.8 (294/738)	0.34	0.91 (0.76–1.10)
Oral antidiabetics, % (*n*/total *n*)	19.6 (253/1292)	18.9 (139/737)	0.63	1.05 (0.83–1.32)
Insulin, % (*n*/total *n*)	20.0 (259/1292)	17.1 (126/737)	0.10	1.22 (0.96–1.54)
Antidepressants, % (*n*/total *n*)	5.2 (67/1294)	6.9 (51/739)	0.11	0.74 (0.51–1.07)
Implanted device, % (*n*/total *n*)	53.1 (690/1299)	46.4 (343/739)	0.004	1.31 (1.09–1.57)
ICD or CRT-D, % (*n*/total *n*)	48.7 (633/1299)	37.6 (278/739)	< 0.001	1.58 (1.31–1.90)

### One-year follow-up

**Fig. 2:**
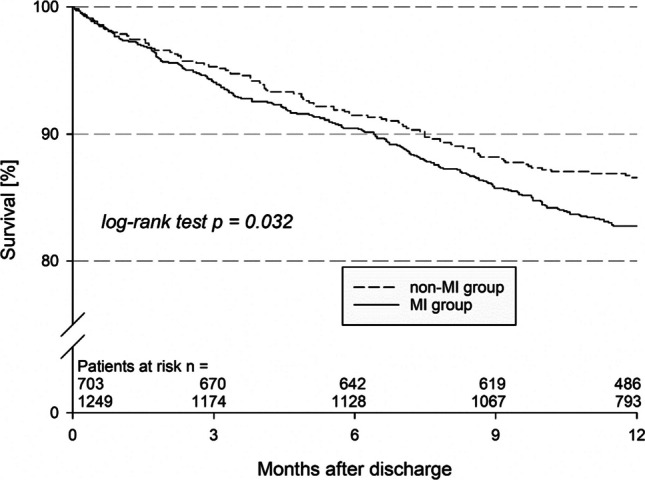
Kaplan–Meier curves showing the mortality of ischaemic HFrEF patients with prior myocardial infarction versus no prior myocardial infarction (dotted line) up to 1 year

**Fig. 3:**
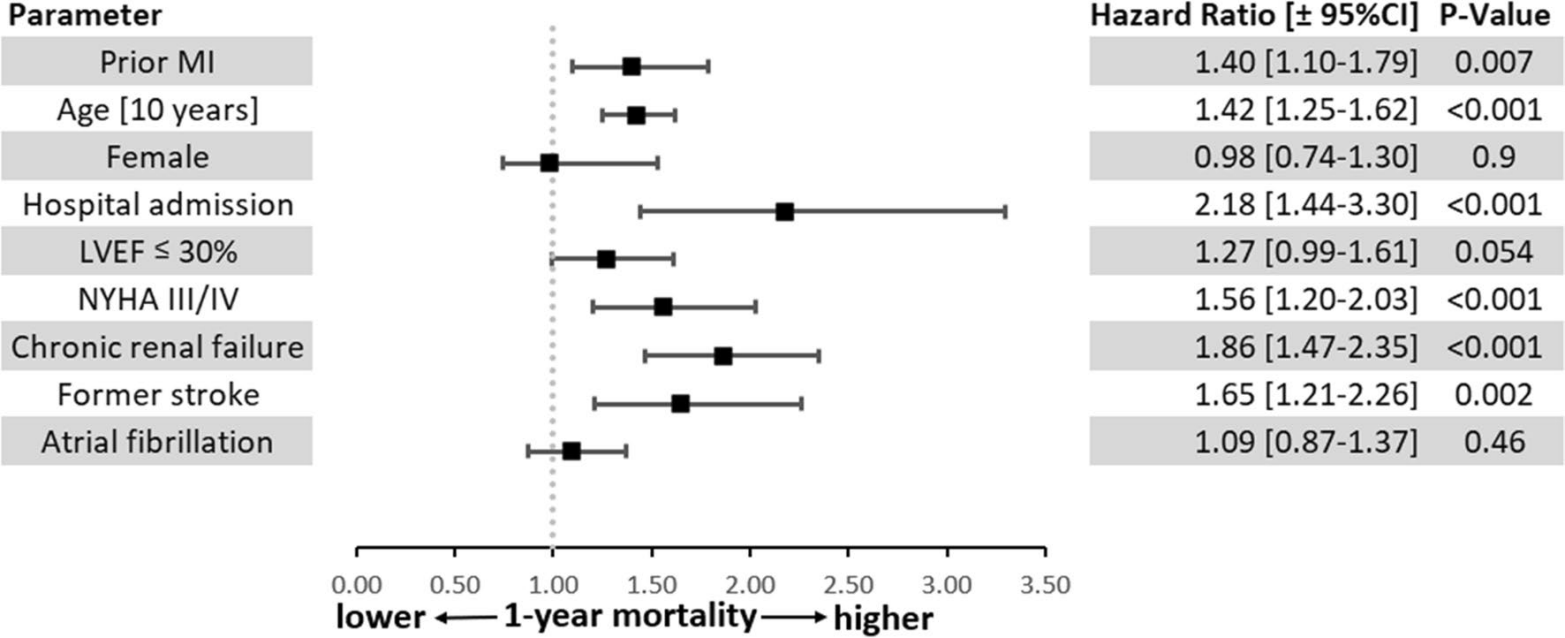
Adjusted 1-year mortality (*n* = 308/1952). Cox regression for 1-year mortality. Inclusion: ischaemic cardiomyopathy who were alive upon discharge from the hospital

The event rates in 1736 patients with complete 1-year follow-up data are given in Table 5. There was a nonsignificant trend toward a higher 1-year all-cause mortality in patients with prior MI in the univariate analysis (17.4 vs. 13.7%, *p* = 0.06). After multivariate adjustment, the 1-year mortality rate was significantly greater in patients with prior MI that was significantly higher compared to that in patients without (adjusted hazard ratio 1.4; 95% confidence interval 1.10–1.79, *p* value 0.007) (Fig. 2). The combination of death, resuscitation or ICD shock occurred more often in patients with prior MI (20.8 vs. 16.4%, *p* = 0.03), whereas there was no difference in nonfatal events between the two groups (Table 5 and Fig. 3). Heart transplantation was planned more than twice as often in patients with prior MI (2.9 vs. 1.4%, *p* = 0.024).

**Table 5:** Events in patients with complete 1-year follow-up (*n* = 1952)

	History of myocardial infarction	No history of myocardial infarction	*p* value	Odds ratio (95% CI)
FU available %, *n*	96.2 (1249/1299)	95.1 (703/741)	0.17	1.35 (0.88–2.08)
FU duration, months, median (IQR)	12.3 (11.6, 13.8)	12.4 (11.9, 13.9)	0.09	
1-year all-cause mortality, %	17.2	13.4	0.03	1.30 (1.02–1.66)
Death or HTX, %	18.0	13.6	0.02	1.35 (1.06–1.71)
Death, myocardial infarction or stroke (MACCE), %	19.4	15.4	0.04	1.27 (1.01–1.60)
Death, resuscitation or ICD shock, %	20.3	15.7	0.02	1.32 (1.05–1.65)
Non-fatal events in survivors				
Resuscitation or ICD shock, % (*n*/total *n*)	4.7 (39/832)	3.3 (16/478)	0.24	1.42 (0.78–2.57)
HTX, % (*n*/total *n*)	1.1 (9/819)	0.2 (1/472)	0.08	5.23 (0.66–41.44)
Myocardial infarction, % (*n*/total *n*)	1.6 (13/824)	1.5 (7/475)	0.88	1.07 (0.42–2.71)
Stroke, % (*n*/total *n*)	1.6% (13/824)	1.7% (8/475)	0.88	0.94 (0.39–2.27)
Severe bleeding, % (*n*/total *n*)	1.5% (8/551)	1.9% (5/269)	0.66	0.78 (0.25–2.40)
NYHA status III/IV, % (*n*/total *n*)	36.7% (219/597)	35.5% (124/349)	0.72	1.05 (0.80–1.38)
Angina pectoris CCS II +	25.7% (104/405)	29.6% (61/206)	0.30	0.82 (0.57–1.19)
Atrial fibrillation, % (*n*/total *n*)	15.2% (91/600)	18.9% (67/354)	0.13	0.77 (0.54–1.08)
ICD, % (*n*/total *n*)	39.1% (300/768)	26.2% (119/455)	< 0.001	1.81 (1.40–2.33)
CRT-D, % (*n*/total *n*)	10.8% (83/768)	18.0% (82/455)	< 0.001	0.55 (0.40–0.77)
PCI, % (*n*/total *n*)	2.1% (14/667)	4.6% (18/389)	0.02	0.44 (0.22–0.90)
CABG, % (*n*/total *n*)	0.9% (6/667)	1.5% (6/389)	0.34	0.58 (0.19–1.81)
HTX planned, % (*n*/total *n*)	4.0% (27/667)	1.3% (5/390)	0.01	3.25 (1.24–8.51)

We hypothesized that patients with insulin-dependent diabetes mellitus could have more silent MIs than patients with insulin-independent diabetes mellitus, so we compared their results with those of all other patients. We found no difference in mortality at follow-up (both 13.4%), in the MACCE rate (15.7 vs. 15.4%) or in nonfatal events in survivors such as resuscitation or ICD shock (both 3.3%). As a result, we had no evidence of additional silent myocardial infarctions in patients with insulin-dependent diabetes mellitus.

### Quality of life

Comparing quality of life on admission and at follow-up, we found that on admission mobility was reduced in almost 50% of the patients with prior MI, and 45% in those without prior MI. Additionally, mobility was ameliorated in both groups 12 months after hospital admission. More than one-fifth of patients in both HFrEF groups had problems caring for themselves, but this rate decreased after 12 months, with a trend toward more patients without prior MI. On admission, usual activities were not possible in approximately 50% of the patients with and without prior MI, but in both groups this rate decreased. Almost 50% of all patients with HFrEF complained of pain or discomfort with no change in follow-up. More than one-fifth of all patients were anxious or depressive with little improvement in patients without prior MI after 12 months (Fig. 5).

## Discussion

The main finding of the present subanalysis of the EVITA-HF registry was that patients with ischaemic HFrEF and a history of prior MI represented a high-risk subgroup within patients with ischaemic HFrEF. We found that more than one-third of patients with ischaemic HFrEF had no history of prior MI. To the best of our knowledge, this is the first comparison of ischaemic HFrEF patients with and without prior MI [1]. A history of prior MI in patients with HF and ischaemic HFrEF in our population was associated with higher all-cause mortality after 1 year, higher event rates and worse health status.

### Extent of CAD

CAD has become the predominant cause of HF with reduced ejection fraction [1, 6]. Previous studies have shown that the extent of coronary artery disease is a better predictor of survival than ischaemic or nonischaemic etiology [8, 9, 14]. In contrast to the STICH trial, other studies have shown a strong association between revascularization and improved survival in patients with a low ejection fraction and significant CAD [14]. Unfortunately, the definition of ischaemic HFrEF is inconsistent [7, 8]. Most of the studies defined ischaemic HFrEF as left ventricular dysfunction with prior MI, percutaneous transluminal coronary angioplasty, coronary artery bypass graft surgery or significant CAD [14]. It has been proposed to reclassify patients with single-vessel disease as non-ischaemic unless they have left main or proximal LAD disease or a history of revascularization or MI [8]; however, this classification system was not widely used. Bart et al. reported that the mortality rate of patients with ischaemic HFrEF and only mild CAD was similar to that of patients in the nonischaemic group [14].

### Medical therapy

With respect to medical heart failure treatment at hospital admission, patients with ischaemic HFrEF and prior MI were treated more often with ACE inhibitors/ARBs, beta-blockers, and mineralocorticoid receptor antagonists (MRAs) than were those without. During hospitalization, the percentage of HF patients who received optimal guideline-directed therapy increased in both groups up to 90% for the use of ACE inhibitors/ARBs and beta-blockers. Notably, MRAs were given to approximately 60% of the patients at discharge, without differences between the two groups compared to the significantly lower percentages at admission.

**Fig. 4:**
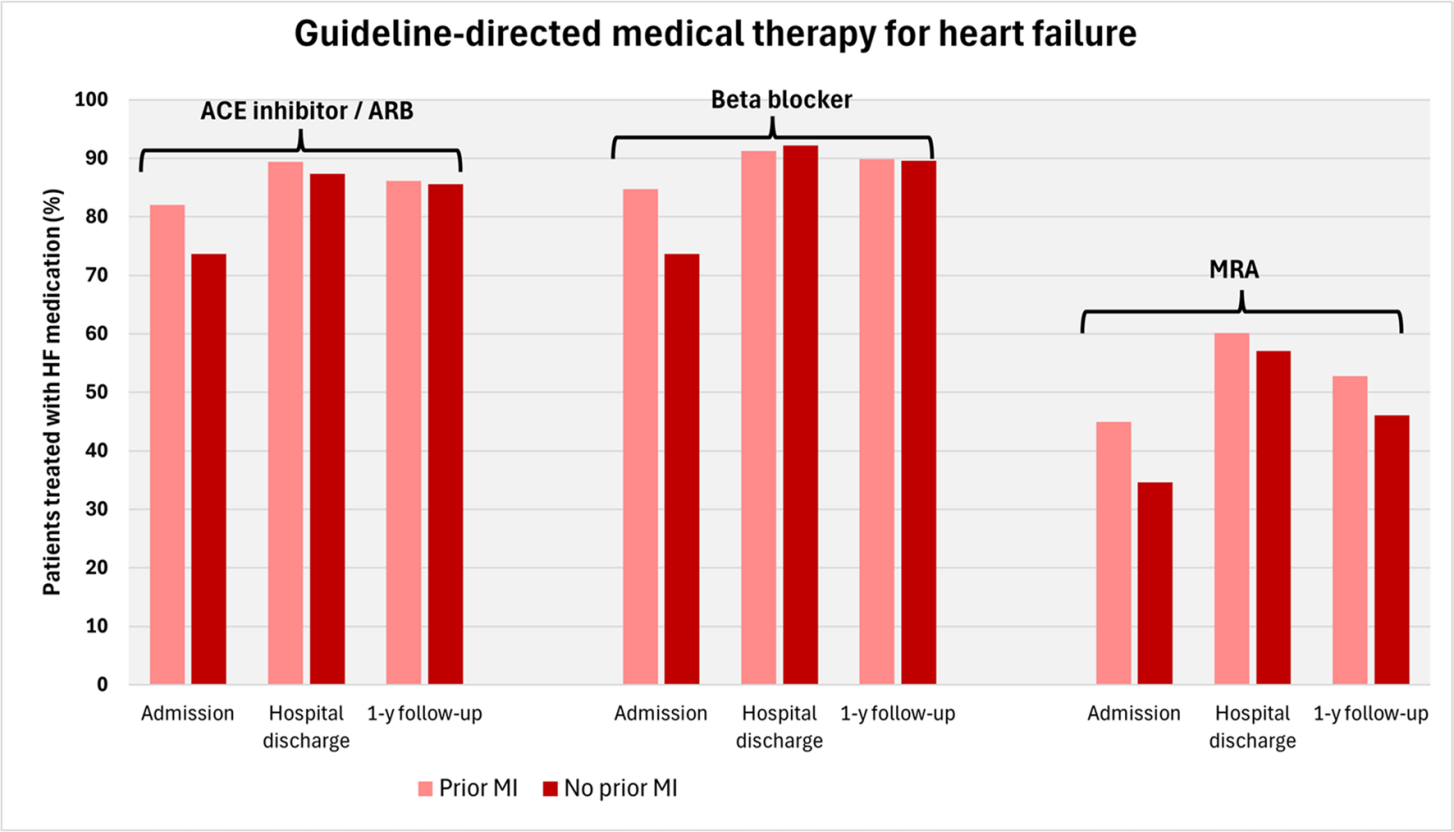
Guideline-directed medical therapy with ACE inhibitors/angiotensin receptor blockers (ARBs), beta-blockers or mineralocorticoid receptor antagonists (MRAs) was administered upon admission, at hospital discharge and the 1-year follow-up. The *y*-axis shows the percentage of patients who were treated

At follow-up, there was a high rate of therapy with ACE inhibitors/ARBs and beta-blockers, whereas the incidence of MRAs was lower than that at admission, but lower than that at discharge (Fig. 4). The reasons for the lower use of MRA compared to ACE inhibitors/ARBs and beta-blockers were already discussed by von Scheidt et al. [13].

Newer heart failure medications such as sacubitril/valsartan or SGLT2 inhibitors were not available at the time of data collection, but in the light of the results of recent milestone studies which have influenced the current heart failure guidelines, these new drugs should be recommended in patients with ischaemic HFrEF, although there was no analysis of patients with and without pMI would benefit identically [3, 15–18]. From preclinical models, there are signs that SGLT inhibitors could reduce infarct size in reperfused ischaemic heart and improve cardiac function [19]. A recent study about SGLT2 inhibitors found that they significantly reduced the inflammatory burden and ameliorated clinical outcomes at 5 years in post-CABG patients with type 2 diabetes mellitus [20].

### Invasive therapy

More than 38% of EVITA-HF patients without pMI were treated with revascularizing therapies during their hospital stay (PCI or CABG), but less than 24% of patients with prior MI were treated. The reasons for this difference can only be speculated. Patients with pMI may already have had invasive therapy before or if they were considered treatment options due to multimorbidity or age. The very recent REVIVED-BCIS2 trial failed to show a beneficial effect of revascularization by PCI among patients with severe ischaemic left ventricular systolic dysfunction who received optimal medical therapy [21]. This study highlights the advancements of pharmacotherapy in heart failure in the past decade. A limitation of this study was that the population consisted of stable, mostly asymptomatic patients selected based on the presence of viable myocardium, so these findings cannot be generalized to all heart failure patients [22].

ICDs for primary prevention reduce all-cause mortality in patients with ischaemic HFrEF [23]. In our population, patients with ischaemic HFrEF and prior MI had more often already had an ICD implanted on admission, but less often had a CRT-D. We found no difference in the rate of ICD shocks between patients with and without prior MI. During the hospital stay, approximately 20% of all patients with ischaemic HFrEF were implanted with an ICD, but the difference between the groups with and without prior MI was not significant.

### Hospital course and follow-up

After 12 months, ischaemic HFrEF patients with prior MI more often sustained the combination of death, resuscitation or ICD shocks than did ischaemic HFrEF patients without MI. This appears plausible because a myocardial scar constitutes an arrhythmogenic substrate and patients are at risk for ventricular fibrillation or ventricular tachycardia [7, 24]. In contrast, patients without prior MI were more often resuscitated during their hospital stay, which cannot be explained by our data. This requires further examination. The mortality rates in our population are comparable to the mortality rates in a large contemporary retrospective US national cohort study that included 68,458 ischaemic HFrEF patients with an LVEF ≤ 35% and a 1-year mortality rate of 12.3% [6]. This rate is higher than that of major ICD RCTs, which reported mortality rates ranging from 7.9 to 9.0% [23]. The higher rates in registries are likely due to the selection of lower risk patients for RCTs [25].

We had no evidence of additional silent myocardial infarctions in patients with insulin-dependent diabetes mellitus.

### Quality of life

**Fig. 5:**
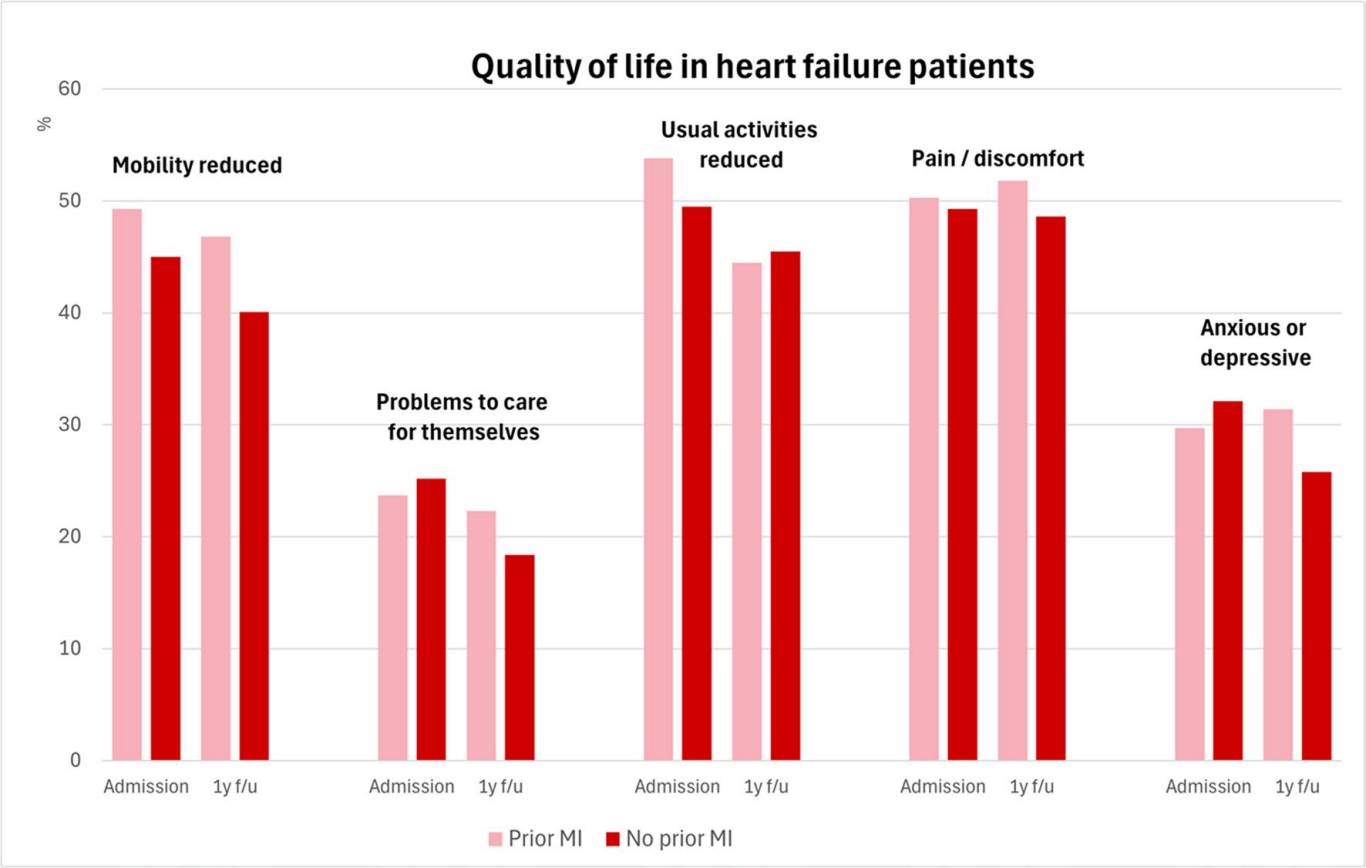
Quality of life in heart failure patients according to the EQ-5D health questionnaire. The *y*-axis shows the percentage of patients with ischaemic HFrEF with reduced mobility, problems caring for themselves, reduced usual activities, pain or discomfort, or anxiety or depression. The bars in light red represent patients with prior MI, and the dark red bars represent those without prior MI

To the best of our knowledge, this is the first study to evaluate the differences in quality of life between ischaemic HFrEF patients with and without prior MI. We showed that after 12 months, patients with prior MI more often had reduced mobility, and they more frequently had worse health status than did those without prior MI (Table 6). Comparing quality of life at admission and at follow-up, we found that in both groups, there was a slight improvement in mobility and in the ability to perform usual activities after 12 months. This could be due to better medical therapy or more intensive general medical care after the last medical contact. Fifty percent of all patients with ischaemic HFrEF complained of pain or discomfort with no change after 12 months. More than one-fifth of all ischaemic HFrEF patients were anxious or depressive with little improvement in patients without prior MI after 12 months (Fig. 5).

**Table 6:** Medication and quality of life in patients with complete 1-year follow-up data

	History of myocardial infarction	No history of myocardial infarction	*p* value	Odds ratio (95% CI)
*n*, (%)	771 (62.8%)	456 (37.2%)		
ACE inhibitor/ARB, % (*n*/total *n*)	86.1% (562/653)	85.6% (321/375)	0.84	1.04 (0.72–1.49)
Beta-blocker, % (*n*/total *n*)	89.9% (587/653)	89.6% (337/376)	0.90	1.03 (0.68–1.56)
MRA, % (*n*/total *n*)	52.8% (345/653)	46.1% (173/375)	0.04	1.31 (1.01–1.69)
Diuretics, % (*n*/total *n*)	79.9% (522/653)	78.1% (293/375)	0.50	1.12 (0.82–1.52)
Amiodarone	2.9% (19/655)	3.5% (13/376)	0.62	0.83 (0.41–1.71)
Quality of life (EQ-5D)				
Health status better compared to 12 months ago, % (*n*/total *n*)	28.9% (185/640)	34.5% (129/374)	0.06	0.77 (0.59–1.01)
Health status unchanged compared to 12 months ago, % (*n*/total *n*)	48.0% (307/640)	49.7% (186/374)	0.59	0.93 (0.72–1.20)
Health status worse compared to 12 months ago, % (*n*/total *n*)	23.1% (148/640)	15.9 (54/340)	0.01	1.6 (1.13–2.26)
Mobility, % (*n*/total *n*)				
Reduced mobility	46.8% (308/658)	40.1% (155/387)	0.03	1.32 (1.02–1.70)
Completely immobile	0.9% (6/658)	0.5% (2/387)	0.48	1.77 (0.36–8.82)
Self-care, % (*n*/total *n*)				
Problems to care for themselves	22.3% (146/655)	18.4% (71/385)	0.14	1.27 (0.92–1.74)
Unable to care for themselves	3.1% (20/655)	1.6% (6/385)	0.14	1.99 (0.79–5.00)
Usual activities, % (*n*/total *n*)				
Reduced	44.5% (292/656)	45.5% (176/387)	0.76	0.96 (0.75–1.24)
No possible to perform any activities	7.3% (48/656)	4.9% (19/387)	0.13	1.53 (0.89–2.64)
Pain/discomfort, % (*n*/total *n*)				
Light or moderate	51.8% (337/651)	48.6% (186/383)	0.32	1.14 (0.88–1.46)
Extreme	8.4% (55/651)	6.8% (26/383)	0.34	1.27 (0.78–2.06)
Anxiety/depression, % (*n*/total *n*)				
Anxious or depressive, % (*n*/total *n*)	31.4% (205/652)	25.8% (99/384)	0.05	1.32 (1.00–1.75)
Extremely anxious or depressive	3.5% (23/652)	2.3% (9/384)	0.29	1.52 (0.70–3.33)

As we could show in the present report, patients with ischaemic HFrEF remain at substantially increased risk of sudden and all-cause death despite advances in cardiac medical and procedural therapies [5, 26]. Hence, determining the best management approach is still challenging [10, 27]. It has been proposed that in the developing era of precision medicine, more detailed phenotyping and genotyping of patients should be performed [1, 3]. Neural network analysis and machine learning may help to identify novel predictive parameters of disease progression and outcomes in the future [28, 29].

## Limitations

The diagnosis of ischaemic HFrEF was based on clinical judgment and investigator-reported aetiology, so misclassifications cannot be fully excluded. In patients with left ventricular dysfunction “out of proportion” to the extent of CAD, the clinical diagnosis of ischaemic HFrEF provides misleading information about expected outcomes. More than 81% of our population were men, so the findings may be not representative for all genders. CMR was not routinely performed in our patients to overcome this limitation. Patients who met the inclusion criteria for patients with chronic systolic HF and an ejection fraction ≤ 40% were considered to have newly diagnosed HF, and patients with HF and a preserved ejection fraction were excluded. All the participating study centres were German tertiary care centres providing the full spectrum of diagnostic and treatment modalities; patients treated in other care settings were not included. Given that EVITA-HF comprises a nonrandomized registry, the contributions of other covariables in addition to the presence of prior MI to reported outcomes cannot be fully excluded. In addition, further information concerning the dosage of medication and the reasons for limited ICD and CRT use should be analysed. Newer heart failure medications such as sacubitril/valsartan or SGLT2 inhibitors were not available at the time of data collection.

## Conclusion

The present subanalysis of the EVITA-HF-registry focused on the influence of prior MI on prognosis in patients with ischaemic HFrEF compared to patients with no prior MI. Approximately two-thirds of patients with ischaemic HFrEF had a history of prior MI, which was associated with increased mortality, increased event rate, and worse health status. Hence, the subgroup of patients with ischaemic HFrEF and prior MI is at an increased risk and deserves special attention.

## Data Availability

Data are available from the corresponding author upon reasonable request.

## Abbreviations

AMI: Acute myocardial infarction
CABG: Coronary artery bypass graft
CAD: Coronary artery disease
CHF: Chronic heart failure (≥ 3 months)
CMP: Cardiomyopathy
CMR: Cardiac magnetic resonance
CRT: Cardiac resynchronization therapy
EVITA-HF: EVIdence based TreAtment in Heart Failure
HF: Heart failure
HFrEF: Heart failure with reduced ejection fraction
ICD: Implantable cardioverter-defibrillator
LAD: Left anterior descending coronary artery
LGE: Late gadolinium enhancement
MI: Myocardial infarction
NICM: Nonischaemic cardiomyopathy
NYHA: New York Heart Association
PCI: Percutaneous coronary intervention
PM: Pacemaker
pMI: Prior myocardial infarction
RCT: Randomized controlled trial
SCD: Sudden cardiac death
